# Correction to “PRMT9 Promotes Hepatocellular Carcinoma Invasion and Metastasis via Activating PI3K/Akt/GSK‐3β/Snail Signaling”

**DOI:** 10.1111/cas.70004

**Published:** 2025-02-12

H. Jiang, Z. Zhou, S. Jin, K. Xu, H. Zhang, J. Xu, Q. Sun, J. Wang, and J. Xu, “PRMT9 Promotes Hepatocellular Carcinoma Invasion and Metastasis via Activating PI3K/Akt/GSK‐3β/Snail Signaling,” *Cancer Science* 109, no. 5 (2018): 1414–1427, https://doi.org/10.1111/cas.13598.

Concerns were raised by a third party regarding overlapping image panels within the article (Figures 2D and 5D). The authors admitted to the image compilation error and were able to provide the raw data of the article. The research integrity office of the authors' institute has investigated the concerns and recommended the publication of a correction. The authors confirm that all the experimental results and corresponding conclusions mentioned in the paper remain unaffected and sincerely apologize for this mistake.

The corrected Figure 5D is below:

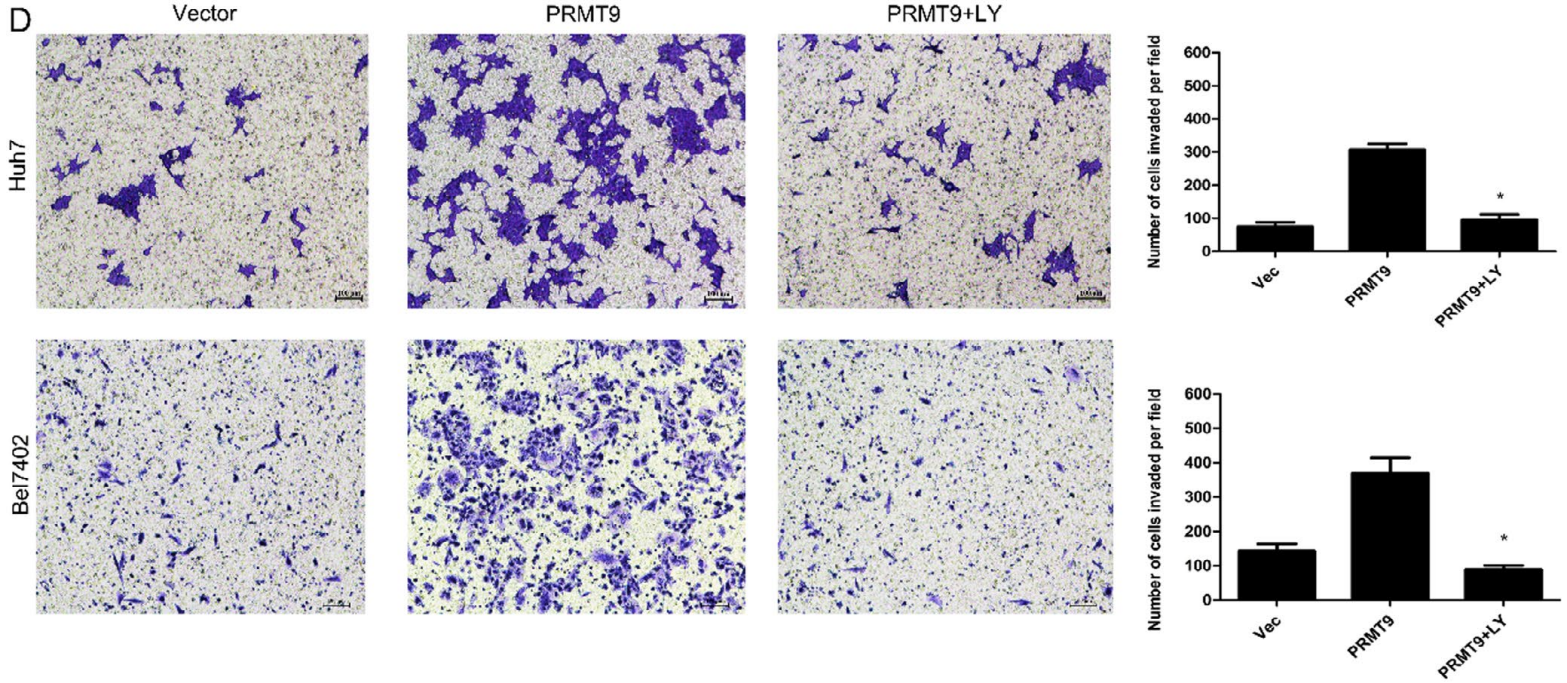

**FIGURE 5** (D) Invasion assays showing that LY294002 inhibits PRMT9‐induced cell invasion.

